# Development of a short and sensitive QuEChERS-SPE-GC-MS method for polycyclic aromatic hydrocarbon (PAH) biomonitoring in *Perna perna* (Linnaeus, 1758) mussels from an oil-impacted tropical estuary

**DOI:** 10.1007/s10661-026-15980-0

**Published:** 2026-10-12

**Authors:** Júlia Vianna de Pinho, Letícia Tessaro, Pamella Talita Silva Melo, Jelmir Craveiro de Andrade, Michael Ribas Celano, Paloma de Almeida Rodrigues, Antonio Eugênio Castro Cardoso de Almeida, Rachel Ann Hauser-Davis, Carlos Adam Conte-Junior

**Affiliations:** 1 National Institute of Health Quality Control, Oswaldo Cruz Foundation, Rio de Janeiro, RJ 21040900 Brazil; 2 Post-Graduate Program in Sanitary Surveillance (PPGVS), National Institute of Health Quality Control (INCQS), Oswaldo Cruz Foundation (FIOCRUZ), Rio de Janeiro, RJ 21040-900 Brazil; 3 Center for Food Analysis (NAL), Technological Development Support Laboratory (LADETEC), Federal University of Rio de Janeiro (UFRJ), Cidade Universitária, Rio de Janeiro, RJ 21941-598 Brazil; 4 Laboratory of Advanced Analysis in Biochemistry and Molecular Biology (LAABBM), Department of Biochemistry, Federal University of Rio de Janeiro (UFRJ), Cidade Universitária, Rio de Janeiro, RJ 21941-909 Brazil; 5 Laboratory of Ecophysiology and Toxicology of Cyanobacteria, Carlos Chagas Filho Biophysics Institute, Federal University of Rio de Janeiro, Rio de Janeiro, 21949-902 Brazil; 6 Fundação Oswaldo Cruz, Instituto Oswaldo Cruz, Laboratório de Avaliação E Promoção da Saúde Ambiental, Rio de Janeiro, RJ Brazil

**Keywords:** Bioindicator, Estuarine bay, Polycyclic aromatic hydrocarbons (PAHs), Green analytical chemistry, Matrix-matched calibration, Pyrogenic sources

## Abstract

The 2019 oil spill in Guanabara Bay, among the most significant in Brazilian history, has reinforced how persistently PAHs move through food webs significant to artisanal fishing communities. However, low-cost tools for tracking these contaminants in key sentinel species, such as bivalves, remain scarce, especially for matrices that resist conventional extraction protocols. In this sense, this study developed a dual-extraction method combining Quick, Easy, Cheap, Effective, Rugged and Safe (QuEChERS) and solid-phase extraction (SPE) clean-up to quantify multiple PAHs in *Perna perna* mussels sampled from Guanabara Bay, Brazil overcoming matrix interference to deliver faster, cheaper and more actionable contamination data for a subsistence food source. A total of 210 mussels were collected across four artisanal fishing communities in Guanabara Bay, a socially and ecologically critical estuary, 2 to 3 years after the spill. The method achieved strong linearity (*r*^2^ > 0.995) and acceptable recovery (51.1–109.0%) for 15 of 18 target PAHs. Only five compounds were quantified above the Limit of Quantification, with special focus on benzo(a)pyrene, with a high-molecular-weight and predominantly pyrogenic signature pointing to a diffuse, combustion-derived background rather than a lingering trace of the spill. This finding highlights the value of the proposed method, of a practical, affordable tool that is extensible to oil-impacted tropical estuaries worldwide for routine PAH biomonitoring.

## Introduction

The intensification of oil exploration activities along the Brazilian coastline has caused considerable environmental concerns, particularly in the southeastern state of Rio de Janeiro (de Oliveira Soares et al., 2020). Guanabara Bay is one of Brazil’s most essential and productive estuaries (Soares-Gomes et al., 2016), albeit highly contaminated by untreated sewage from domestic and industrial sources and by petroleum platforms that surround the bay. In this sense, oil extraction and refining activities along the Brazilian coastline represent a persistent source of polycyclic aromatic hydrocarbon (PAH) contamination in coastal and estuarine environments. This has led to concerns regarding the adequacy of current environmental legislation and the effectiveness of current safety protocols (Umar et al., 2021). This is especially true for the treatment of contaminants such as polycyclic aromatic hydrocarbons (PAHs), which are some of the most harmful compounds discharged during oil spills, due to their persistence and bioaccumulation and mutagenic and carcinogenic potential (Szopińska et al., 2019). The 2019 oil spill contaminated over 3000 km of coastline across nine northeastern states of the country and reached as far south as the northern coast of Rio de Janeiro state (de Figueiredo Mendes et al., 2021). Tar balls chemically linked to the 2019 spill continued to reach Brazilian beaches years later, marking a long-term environmental legacy (Pereira et al., 2023), similar to the Prestige spill, where PAH contamination persisted for years after the spill event (Fernandez-Tajes et al., 2011).

Polycyclic aromatic hydrocarbons are produced mainly during the incomplete combustion of organic materials such as fossil fuels, tobacco and plant waste (Kravchenko & Minkina, 2025) but also originate from petrogenic sources associated with oil spills, chronic vessel traffic, and industrial discharges (Cabral et al., 2022). Human exposure to PAHs is continuous and occurs mainly through inhalation of airborne particles and the consumption of polluted water and contaminated food (Hatami Manesh et al., 2021). The accumulation of these compounds in fatty tissues is chronic, due to their lipophilic nature, which leads to increased biomagnification risks along food chains (Zelinková & Wenzl, 2015).

Monitoring of PAH contamination in marine environments is successfully carried out by analysing bioindicators, such as bivalve molluscs, including *Perna perna* (Linnaeus, 1758) mussels. These animals are widely recognized as valuable bioindicators due to their filter-feeding behaviour, which makes them highly susceptible to contaminant ingestion and lipophilic contaminant accumulation (Hatami Manesh et al., 2021), as well as their tolerance to a broad range of environmental conditions (Yoshimine & Carreira, 2012; Degger et al., 2011). The suitability of *P. perna* as a PAH biomonitor in GB has been proven experimentally, in which PAH burdens in mussels collected from the Rio-Niterói Bridge pillars increased two to fivefold following a fuel oil spill in 2000, with concentrations closely tracking changes in the surrounding water column (Francioni et al., 2005). In Brazil, *Perna perna* mussels are economically significant to several fishing communities, indicating the need for contaminant monitoring efforts (Francioni et al., 2007b). However, despite technological advances, adequately detecting PAHs in biological samples remains challenging.

Gas chromatography coupled with mass spectrometry (GC-MS) is a highly sensitive and selective analytical method but often limited by biological sample complexity and high operational costs (Aresta & Zambonin, 2023). When combined with the Quick, Easy, Cheap, Effective, Rugged and Safe (QuEChERS) approach, originally developed for pesticide analysis, GC-MS offers a simplified, cost-effective extraction pathway that aligns with green chemistry principles by reducing hazardous solvent consumption and analytical waste (Anastassiades et al., 2003; Jalili et al., 2020). Solid-phase extraction (SPE) is commonly employed downstream of QuEChERS as a clean-up step, removing co-extracted lipids and matrix interferents prior to GC-MS analysis, thereby improving chromatographic resolution and analyte selectivity in lipid-rich biological matrices such as mussel tissue. Adapting this method to lipid- and protein-rich biological matrices, however, requires careful optimization and thorough validation to ensure adequate recovery and selectivity across a wide range of PAH (Zelinková & Wenzl, 2015; Aresta & Zambonin, 2023). Imputation strategies for non-detected values, such as substitution by LOD/2 as recommended by the World Health Organization (WHO, 1995), further support the statistical robustness of monitoring datasets (Helsel, 2006) regarding PAH monitoring in biological samples.

In recent years, QuEChERS-based extractions have been validated for subsequent PAH and other contaminant analyses across a wide range of matrices, including fish (Duedahl-Olesen et al., 2020; Forsberg et al., 2011; Kao et al., 2012; Khorshid et al., 2015; Ramalhosa et al., 2009), sediment (Salem et al., 2016), soil (Fahd et al., 2019), plant materials (Hengel et al., 2019; Rodrigues et al., 2019; Shin et al., 2021; Słowik-Borowiec et al., 2022), milk-related matrices (Kim et al., 2017; Yan et al., 2021), and beverages (Silva et al., 2018). Its application to biological, animal-derived tissue, however, remains comparatively limited, with scarce applications to bivalve tissues. Complementary extraction strategies for lipid-rich tissues have relied on matrix solid-phase dispersion (Fernández-González et al., 2010), accelerated solvent extraction with gel-permeation clean-up (Yusà et al., 2005), or supercritical- and pressurized-fluid extraction (Ali & Cole, 1998; Benner, 1998; Schantz et al., 2012), rather than QuEChERS-SPE, even though the QuEChERS approach reduces both cost and analysis times. For example, relative to pressurized solvent extraction, QuEChERS lowered the cost per extraction roughly fivefold and increased sample throughput tenfold (Albinet et al., 2013), a combination directly relevant to laboratories seeking to expand PAH biomonitoring capacity without proportional increases in operating costs.

In this context, this study employed a QuEChERS-SPE-GC-MS integrated methodology for the identification and quantification of PAHs in *Perna perna* mussels sampled roughly 2 years after the 2019 oil spill from the coast of southeastern Brazil, combining optimized extraction and clean-up steps with WHO-recommended imputation techniques to improve the statistical treatment of censored environmental data.

## Material and methods

### Chemicals

A certified PAH standard mixture containing the European priority pollutant (16 PAH) was purchased from Restek (Bellefonte, PA, USA). Individual naphthalene and phenanthrene standards were also included to ensure comprehensive coverage of PAHs of environmental interest. With this, a total of 18 PAHs were analysed including benz(a)anthracene, benzo(a)pyrene, benzo(b)fluoranthene, benzo(c)fluorene (3,4-benzofluorene), benzo(g,h,i)perylene, benzo(j)fluoranthene, benzo(k)fluoranthene, chrysene, cyclopenta(c,d)pyrene, dibenzo(a,e)pyrene, dibenz(a,h)anthracene, dibenzo(a,h)pyrene, dibenzo(a,i)pyrene, dibenzo(a,l)pyrene, indeno(1,2,3-cd)pyrene, 5-methylchrysene, naphthalene, and phenanthrene. All stock and working solutions were prepared using the mixture solution and dichloromethane (DCM) HPLC-grade and stored at − 20 °C. n-Hexane (HEX) and acetonitrile (ACT), used in the extraction and clean-up processes, both HPLC-grade, were purchased from TediaBrazil (RJ, Brazil) and Florisil (particle size: 0.150–0.250 mm) was obtained from Merck.

### Study area

**Fig. 1:**
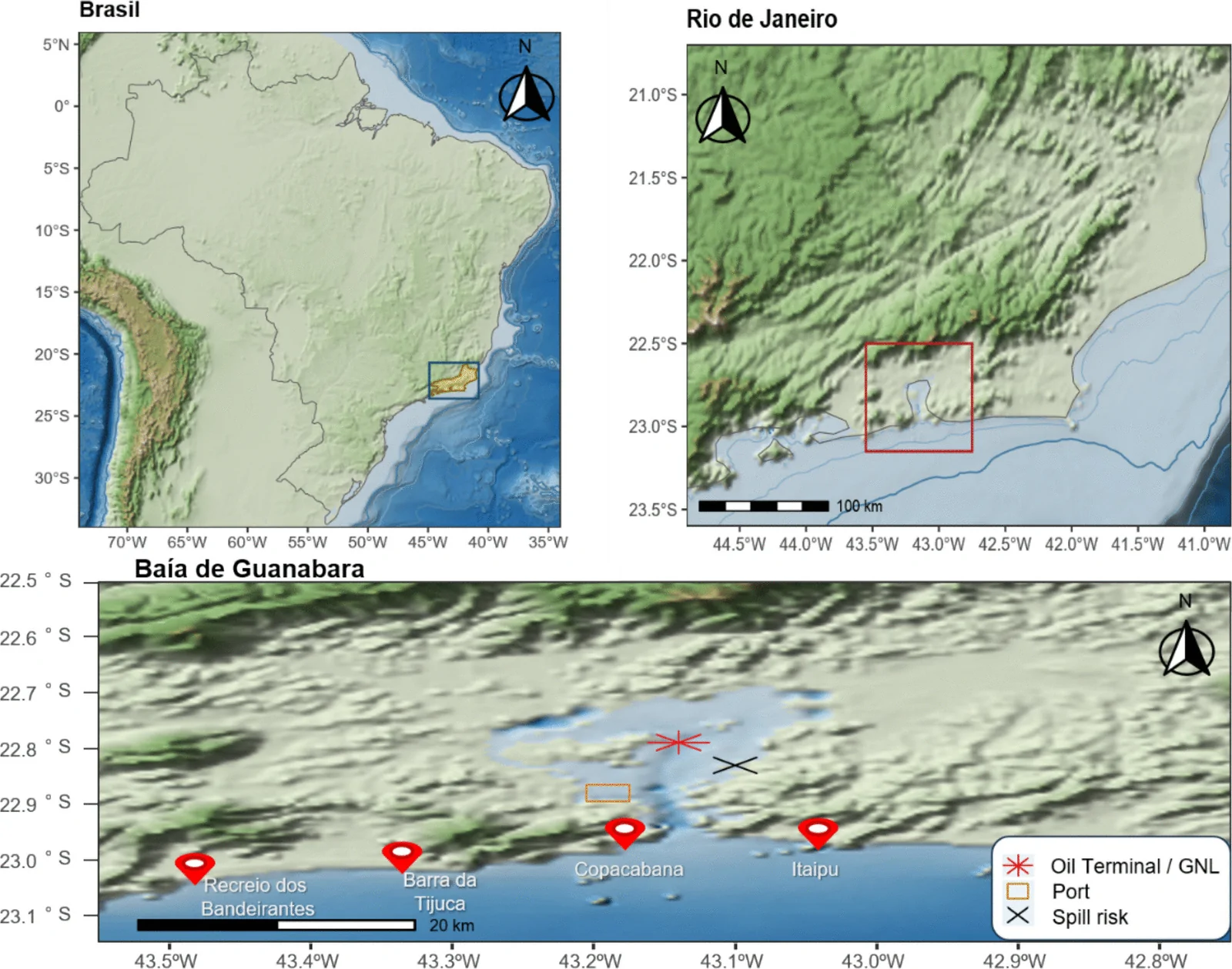
Location of mussel sampling sites in Rio de Janeiro, southeastern Brazil. Red pins indicate the four sampling sites (Itaipu, Copacabana, Barra da Tijuca, and Recreio dos Bandeirantes)

Mussels were sampled from four artisanal fishing sites (Itaipu, Copacabana, Barra da Tijuca and Recreio dos Bandeirantes, Fig. 1) in the coastal zone adjacent to Guanabara Bay (GB) almost 2 years after the 2019 oil spill, a time window suited to assessing medium-term PAH persistence in coastal marine biota, beyond the acute-phase response documented in the immediate aftermath of the disaster. The artisanal fishing sites were chosen for their representativity of artisanal fisheries in the region. Guanabara Bay is the second-largest bay on the Brazilian coast (~ 384 km^2^), bordered by a metropolitan region of over 16 million people, in the state of Rio de Janeiro (Fistarol et al., 2015). Its northwestern sector concentrates much of the region’s petrochemical infrastructure, including refineries and oil-storage terminals, making GB one of South America’s most heavily oil-impacted estuarine systems (Soares-Gomes et al., 2016). This industrial and urban pressure has been repeatedly documented through high PAH burdens in the bay’s sediments, reflecting both chronic petrogenic inputs from oil handling and pyrogenic inputs from urban and vehicular combustion (Christensen et al., 2010). The selected sites, sustaining economically and culturally important artisanal mussel fisheries, offer a relevant setting for assessing PAH persistence and bioavailability in coastal food webs in the years following the 2019 spill.

### Sampling, dissection, and processing

Mussels (~ 20 individuals per pool; 210 individuals in total) were purchased whole from artisanal fishers at the four fishing colonies following the point sampling method, across four seasonal campaigns (summer, autumn, winter and spring) between September 2021 and June 2022. Sampling was attempted at all four sites in each of the four campaigns (16 planned site-season combinations); however, no individuals were available for collection at Recreio dos Bandeirantes in winter and spring, Copacabana in spring, and Barra da Tijuca in summer and autumn, due to the absence of harvestable mussels during those periods. Consequently, 11 pooled samples were obtained and analysed, distributed as follows: Itaipu (4 pools, all seasons), Copacabana (3 pools: summer, autumn, winter), Barra da Tijuca (2 pools: winter, spring), and Recreio dos Bandeirantes (2 pools: summer, autumn). Specimens were immediately placed on ice and transported to the laboratory, where they were identified at the species level, measured with and without shells, and classified according to processing state (“fresh, in-shell” or “pre-cooked, shucked”). Specimens were then dissected, and each pool (~ 20 individuals per site per campaign) was homogenized and divided into three replicate aliquots (1.0000 ± 0.0100 g each), stored at −20 °C in sterile 50 mL polypropylene screw-capped tubes until analysis, with each pool analysed in triplicate.

### Polycyclic aromatic hydrocarbon determinations

**Fig. 2:**
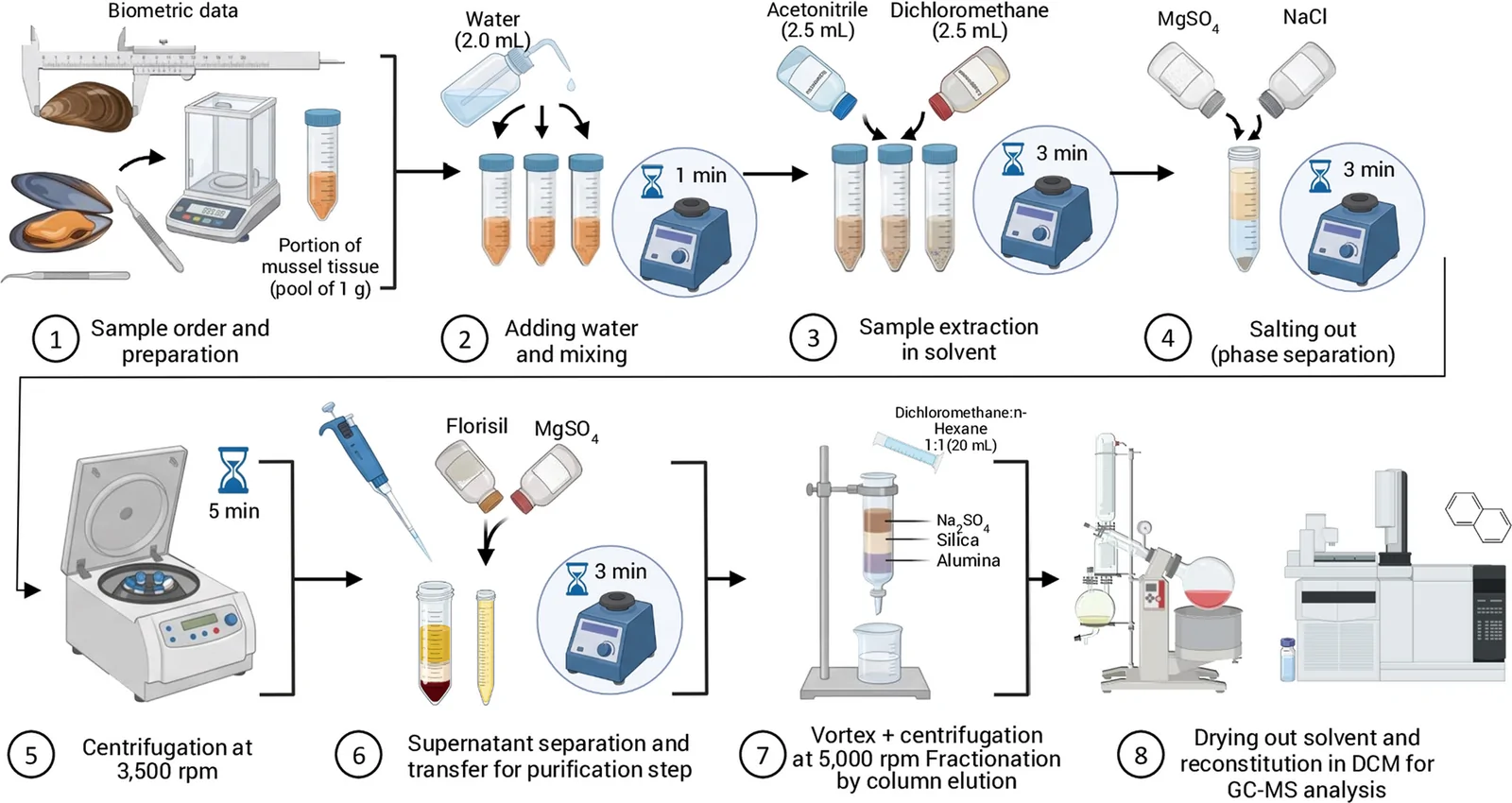
Schematic representation of the modified QuEChERS extraction procedure (adapted from Anastassiades et al., 2003) for PAH analysis in *Perna perna* mussels. The workflow includes sample homogenization, solvent extraction using a 1:1 acetonitrile and dichloromethane mixture, salt addition, and centrifugation

Mussel tissues (*n* = 210 individuals, pooled in triplicate per site) were homogenized and divided into three replicate aliquots (1.0000 ± 0.0100 g each). The aliquots were fortified with the 18 target PAHs (benzo[b]fluoranthene, benzo[c]fluorene, benzo[g,h,i]perylene, benzo[j]fluoranthene, benzo[k]fluoranthene, chrysene, cyclopenta[c,d]pyrene, dibenzo[a,e]pyrene, dibenz[a,h]anthracene, dibenzo[a,h]pyrene, dibenzo[a,i]pyrene, dibenzo[a,l]pyrene, indeno[1,2,3-cd]pyrene, 5-methylchrysene, phenanthrene, and naphtalene), prepared as an acetonitrile stock solution at three concentration levels (1.00, 1.50 and 2.00 ng g⁻^1^), and allowed to equilibrate before freezing and subsequent extraction. Each replicate was homogenized for 1 min with 2 mL of ultrapure water using a vortex mixer (Thermo Scientific) and vortexed for 3 min in a 1:1 combination of acetonitrile and dichloromethane at 2500 rpm, selected to improve the extraction efficiency of lipophilic compounds such as PAHs. Extraction salts (2.0000 g MgSO_4_ and 1.0000 g NaCl) were added to the homogenized samples and vortexed for an additional 3 min at 2000 rpm. The mixtures were then centrifuged for 5 min at 3500 rpm at 4 °C (Thermo Scientific) (Fig. 2). All concentrations reported in this study are expressed as wet weight (fresh tissue).

Each sample solvent layer was added to tubes containing clean-up salts (0.1000 g of Florisil and 0.4500 g of MgSO_4_). After another centrifugation and vortexing step, the supernatants were separated and, to ensure complete clean-up, PAHs were retained on a chromatographic column containing 3.0000 g of deactivated 5% silica gel, 2.000 g of deactivated 2% alumina, and approximately 1.0000 g of anhydrous Na_2_SO_4_ for moisture retention. The PAHs were then eluted from the column using 20 mL of DCM and HEX (1:1). Eluates were concentrated using a rotaevaporator (Fisatom, model 801) and adjusted to 1 mL with DCM for the GC-MS analysis.

### GC-MS conditions

The GC-MS analysis was performed on a Shimadzu QP2010 Plus system (Shimadzu Corporation, Japan) equipped with a DB-5MS capillary column (30 m × 0.25 mm i.d., 0.25 μm film thickness), using helium (99.999% purity) as carrier gas. The oven temperature programme is detailed in Table 1. Samples (1 μL) were injected in splitless mode at 280 °C to maximise sensitivity, with ionization by electron impact (70 eV) at a source temperature of 200 °C. Data acquisition was performed in Selected Ion Monitoring (SIM) mode across three channels, spanning 5.00–76.00 min. The specific time windows and monitored m/z ratios per channel are also given in Table 1.

**Table 1:** Gas chromatography mass spectrometry conditions used for the analysis of Polycyclic Aromatic Hydrocarbons in *Perna perna* mussel samples

Gas chromatography conditions				
Step	Temperature increase rate		Target temperature	Hold time
1	*-*		80°C	2.00 min
2	20°C/min		180°C	0.00 min
3	5°C/min		230°C	2.00 min
4	2°C/min		300°C	2.00 min
5	1°C/min		310°C	0.00 min
Mass spectrometry conditions				
Time interval (min)	Acquisition mode	Channel 1 (m/z)	Channel 2 (m/z)	Channel 3 (m/z)
5.00–21.00	Selected ion monitoring	216	-	-
21.00–25.00	Selected ion monitoring	216	226	228
25.00–28.00	Selected ion monitoring	242	242	242
28.00–33.00	Selected ion monitoring	252	252	252
33.00–38.00	Selected ion monitoring	252	252	252
38.00–50.00	Selected ion monitoring	276	278	278
50.00–58.00	Selected ion monitoring	302	302	302
58.00–76.00	Selected ion monitoring	302	302	302

### Analytical performance assessment

The analytical performance of the QuEChERS-SPE-GC-MS method was assessed to verify its fitness for purpose in the context of environmental biomonitoring of PAHs in marine bivalve tissues. Performance criteria were established primarily in accordance with USEPA Method 8270D guidelines for semi-volatile organic compound determination in environmental matrices (USEPA, 2007), complemented by WHO recommendations for analytical quality assurance in environmental monitoring programs (WHO, 1995). European Commission Regulation 2007/333/EC (European Commission, 2007) and CODEX Alimentarius document CAC/GL 71–2009 (CODEX, 2009) were additionally considered as reference frameworks for recovery and precision criteria, given their established use in PAH method performance benchmarking in biological matrices.

### Selectivity

Method selectivity was evaluated using mussel samples enriched with a standard mixture of 18 HPAs to assess potential interferences in the chromatographic regions of interest where each HPA is expected to elute (European Commission, 2007; USEPA, 2007). Selectivity was considered adequate when target PAHs could be unambiguously identified by retention time and confirmed ion ratios in the presence of the mussel matrix, without significant interference from co-extracted compounds.

### Matrix effects

Potential matrix effects were assessed by comparing the chromatographic responses of PAH standards prepared in pure solvent with those obtained from matrix-matched calibration solutions prepared in mussel extract. The signal enhancement or suppression induced by the biological matrix was quantified for each compound using the *F*-test, which evaluated the statistical significance of differences between the slopes and intercepts of solvent and matrix calibration curves (CODEX, 2009; USEPA, 2007). Matrix-matched calibration was adopted as the default quantification strategy where significant matrix effects were identified.

### Limits of detection (LOD) and quantification (LOQ)

The Limit of Detection (LOD) and Limit of Quantification (LOQ) were determined according to European Commission (European Commission, 2007) and CODEX Alimentarius (CODEX, 2009) guidelines, from triplicate samples containing increasing PAH concentrations (1.00, 1.50 and 2.00 ng mL^−1^). The LOD was defined as the minimum concentration producing a signal three times greater than the background noise, while the LOQ was determined as the lowest concentration providing reproducible results, with a signal ten times greater than the background noise. The instrumental LOD (LODi) and LOQ (LOQi) were determined as follows:

1 $${LOD}_{i}=3.3\times \frac{s}{S}$$

2 $${LOQ}_{i}=10\frac{s}{S}$$

where S is the angular coefficient of the slope and *s* is the standard deviation of the linear coefficient.

### Recovery and precision

Quantifications were performed using matrix-matched calibration rather than deuterated internal standards, accounting for matrix-induced signal enhancement or suppression in mussel extracts. One calibration-curve point and procedural blanks were included in every analytical batch to monitor instrument stability; all calibration, injection, and sample analyses were completed within a 2–3-week period to minimize instrumental drift. Method performance was further documented through spiking experiments at three concentration levels, providing recovery and precision data as indicators of extraction consistency. Precision was assessed in accordance with Commission Regulation 2007/333/EC (European Commission, 2007), by result repeatability at the three concentrations of PAHs, using three replicates at each concentration level and calculated based on variations between replicates (Eq. 3).

3 $$Recovery \,\left(\mathrm{\%}\right)=\left(Concentration \,after\, extraction/Spiked \,concentration\right)\times 100$$

To assess accuracy, recoveries were calculated by comparing the nominal PAH concentrations added to each mussel sample to the measured concentrations in the enriched samples. The average recovery of all PAHs was determined for each concentration level, and accuracy was deemed acceptable when relative standard deviations (RSD) values remained below 20% at each fortification level, following European Commission (2007) and CODEX Alimentarius (CODEX, 2009) standards for method validation of complex biological matrices.

### Data analysis

Method validation parameters were assessed statistically. Linearity was evaluated from the coefficient of determination (*r*^2^) of calibration curves built in both pure solvent and matrix-matched conditions; reproducibility between the two curve models was assessed for each compound using the *F*-test, to determine whether matrix composition significantly altered curve slope or intercept. Precision was expressed as the coefficient of variation (CV) among triplicate aliquots of each pooled sample, and accuracy was assessed through recovery experiments at three fortification levels (1.00, 1.50, and 2.00 ng g^−1^).

For the environmental dataset, values below the LOD were replaced by LOD/2, following WHO (1995) guidance for censored environmental datasets; compounds with non-detection rates exceeding 60% were excluded from imputation and treated as non-quantifiable (Helsel, 2006; WHO, 1995). Data normality was assessed using the Shapiro–Wilk test, and homogeneity of variances was evaluated using Cochran’s test. As PAH concentrations were non-normally distributed across all sampling groups, non-parametric tests were applied throughout. Differences in PAH concentrations across sampling sites and seasons were assessed using the Kruskal–Wallis test, followed by pairwise comparisons using the Mann–Whitney test. Reproducibility between calibration curve models was assessed using the *F*-test. Statistical significance was set at *α* = 0.05. All analyses were performed in R software version 4.4.2 (R Core Team, 2020). Consumer health risk assessment indices including Estimated Daily Intake (EDI), Hazard Quotient (HQ), Toxic Equivalency Quotient (TEQ) and Incremental Lifetime Cancer Risk (ILCR) were considered within the analytical scope of this study. However, as the measured PAH concentrations in *P. perna* were predominantly below the method LOQ, the application of the risk calculations and render has resulted indices unreliable.

## Results and discussions

### Chromatographic analysis

**Fig. 3:**
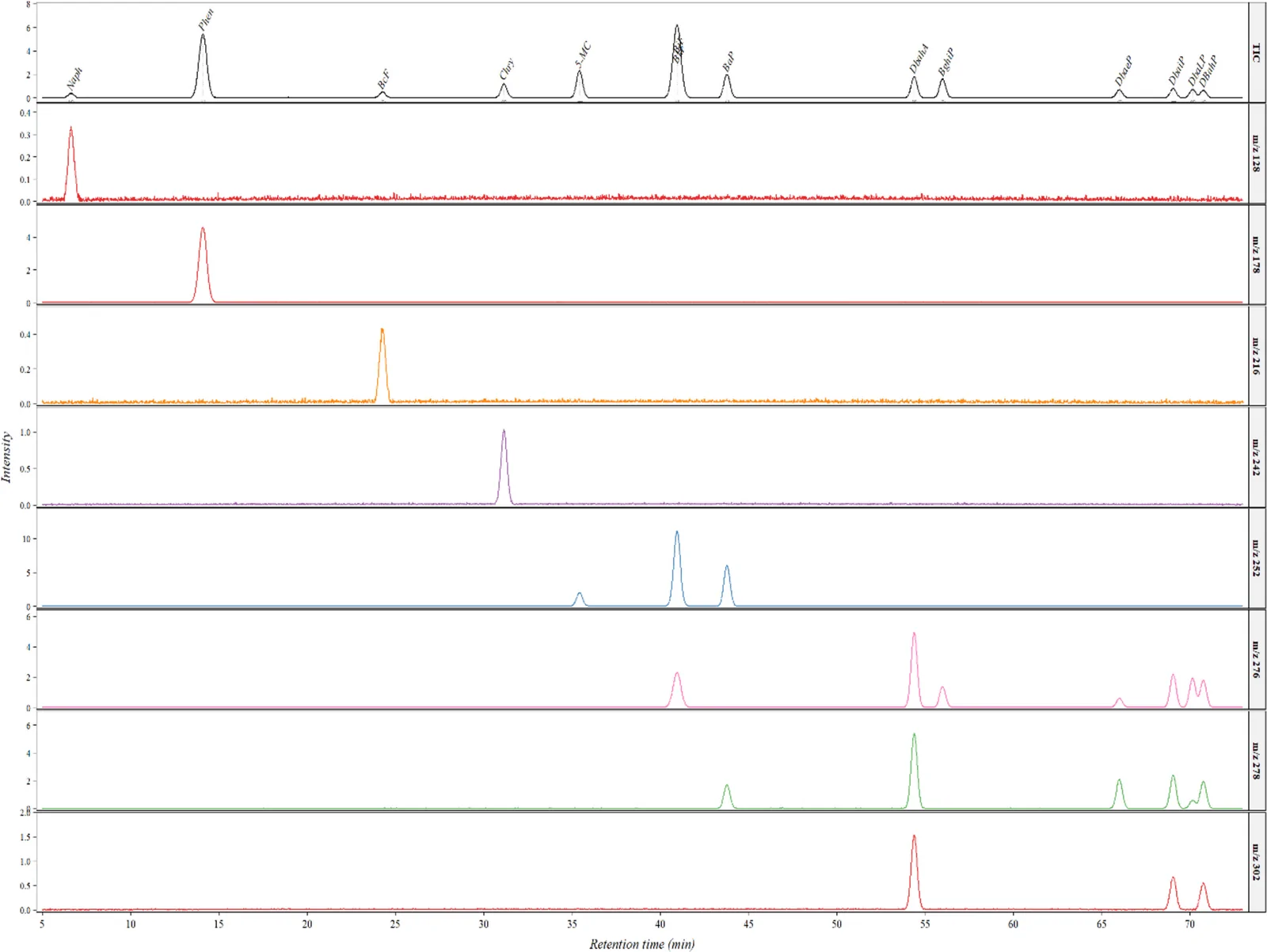
Total ion chromatogram (TIC) and selected ion chromatograms (SIC) of PAHs analysed by gas chromatography-mass spectrometry (GC-MS) in selected ion monitoring (SIM) mode. The TIC (black) represents the sum of all ionic signals, while individual ion channels correspond to the quantification ions (m/z) of each compound: m/z 128 (naphthalene, Naph; red), m/z 178 (phenanthrene, Phen; dark red), m/z 216 (benzo(c)fluorene, BcF; orange), m/z 242 (chrysene, Chry; purple), m/z 252 (5-methylchrysene, 5-MC; benzo(j)fluoranthene, BjF; and benzo(k)fluoranthene, BkF; blue), m/z 276 (benzo(ghi)perylene, BghiP; dibenzo(a,e)pyrene, DbaeP; dibenzo(a,i)pyrene, DbaiP; pink), m/z 278 (benzo(a)pyrene, BaP; dibenzo(a,h)anthracene, DbahA; dibenzo(a,l)pyrene, DbaLP; green), and m/z 302 (indeno(1,2,3-cd)pyrene, IPi; dibenzo(a,h)pyrene, DBahP; red)

The identification and quantification of PAHs in biological matrices such as mussel tissue present significant analytical challenges, including overlapping chromatographic peaks and structural similarities among congeners. Figure 3 presents a representative GC-MS chromatogram of the 18 target PAHs analysed *P. perna* mussel extracts. Each peak was assigned based on retention time matching and ion ratio confirmation against a certified standard mixture. Baseline separation of all 18 target compounds was achieved within the established temperature programmed, demonstrating that the combined QuEChERS-SPE extraction effectively isolated PAHs from co-extracted matrix components, preserving both chromatographic resolution and signal integrity.

Among the detected PAHs, Benzo(a)pyrene (BaP) was identified with a clearly resolved peak around 14.0 min, highlighting the method's ability to separate it from other interfering compounds present in this complex matrix. This is one of the most toxic PAHs and is often used as a reference in international regulations, such as the European Union, which sets a maximum limit of 5 ng g^−1^ in seafood by Regulation 2007/333/EC due to its carcinogenic potential (European Commission, 2007; CODEX, 2009). The presence of this compound can be associated with anthropogenic pollution sources, such as fossil fuel and biomass burning (Cabral et al., 2022). The optimized GC-MS method combined with QuEChERS extraction detected four PAHs at concentration levels compatible with international regulatory limits.

The QuEChERS approach was originally developed for pesticide residues in plant matrices (Anastassiades et al., 2003) but has since been widely extended to persistent organic pollutants, including PAHs, in biological tissue, owing to its simplicity, speed, and minimal solvent and reagent use (Rejczak & Tuzimski, 2015; Vaclavik et al., 2018). Its combination with SPE clean-up has proven particularly effective for lipid-rich matrices such as fish muscle, breast milk, and mussel tissue, where excess co-extracted lipids are the primary source of chromatographic interference and matrix effects (Cloutier et al., 2017; Cunha et al., 2010). Cloutier et al. (2017) specifically validated a QuEChERS-based workflow for PAHs, PCBs, PBDEs and PCDD/Fs in blue mussel and salmon tissue, indicating that this extraction strategy is already an established, though not yet standardized, choice for bivalve biomonitoring.

Beyond extraction efficiency, QuEChERS-based methods offer substantial gains in cost and turnaround time relative to conventional approaches such as Soxhlet extraction. A recent QuEChERS-GC-MS method, for example, developed for PAHs in human adipose tissue shortened analysis time and lowered extraction costs relative to SPE-based protocols, providing a simpler and greener alternative that dispenses with a clean-up step (Sousa et al., 2023). Another optimized QuEChERS protocol for PAHs, this time for aerosol particles, reported a 97% reduction in per-sample cost relative to Soxhlet extraction (Jing et al., 2023). Comparable recovery gains have also been reported for biological matrices, with higher PAH recoveries employing QuEChERS (84–101%) compared to ultrasound-assisted extraction (54–68%) across porcine, avian, and fish tissues (Krakowska-Sieprawska et al., 2022). These efficiency gains carry particular weight in the context of routine environmental biomonitoring in resource-limited settings, where sophisticated analytical infrastructure is frequently unavailable or unaffordable (Islam et al., 2022).

### Analytical performance

#### Selectivity

Chromatographic and statistical selectivity were evaluated together (Fig. 3; Table 2). Most target compounds resolved as distinct, baseline-separated peaks, with linearity holding at *r*^2^ > 0.95 across the method range for nearly the entire panel. The *F*-test reinforced this, comparing solvent and matrix calibration curves and showing no statistically significant difference for any compound (*p* > 0.05), including high-molecular-weight (HMW) PAHs that are typically the most prone to matrix interference, such as phenanthrene and BjF (*F* = 3.30 × 10^4^, *p* = 0.990).

**Table 2:** Gas chromatography mass spectrometry mussel polycyclic aromatic hydrocarbon calibration parameters

Compound	Equation		*r*^2^		*F* value	*p* value
	Instrumental range	Method range	Instrumental range	Method range		
5-MC	*y* = 2666.683 × *x* + −128.817	*y* = 13,725.4 × *x* + 1489.9	0.990	0.963	627E + 03	0.999
BaA	*y* = 9800.024 × *x* + −572.226	*y* = 49,844.4 × *x* + 1304.2	0.992	0.962	301E + 03	0.999
BaP	*y* = 8685.049 × *x* + −289.701	*y* = 105,440.743 × *x* + 3446.867	0.972	0.962	817E + 03	0.998
BcF	*y* = 2733.244 × *x* + 93.494	*y* = 15,659.8 × *x* + 68.3	0.993	0.978	182E + 03	0.999
BghiPer	*y* = 5439.976 × *x* + −333.774	*y* = 59,713.029 × *x* + 2345.2	0.994	0.982	519E + 05	**0.698**
BjF	*y* = 74,323.268 × *x* + −3940.232	*y* = 238,103.324 × *x* + −35,940.919	0.809	0.976	330E + 04	0.990
BkF	*y* = 14,404.707 × *x* + −752.543	*y* = 77,896.4 × *x* + 39,892.8	0.974	0.977	287E + 03	0.999
CCP	*y* = 1892.78 × *x* + 96.78	*y* = 21,254.6 × *x* + −1701.7	0.997	0.994	327E + 03	0.999
Chry	*y* = 11,617.854 × *x* + −669.146	*y* = 65,171.143 × *x* + −18,489.667	0.993	0.959	382E + 02	0.999
DbaePy	*y* = 4516.366 × *x* + −220.384	*y* = 101,907.027 × *x* + −36,756.243	0.993	0.979	407E + 03	0.999
DbahA	*y* = 3536.073 × *x* + −239.677	*y* = 39,940.229 × *x* + −3065.733	0.992	0.990	116E + 02	0.999
DbahPy	*y* = 3661.8 × *x* + 581.5	*y* = 104,821.676 × *x* + −26,187.081	0.995	0.972	433E + 01	0.999
DbaiPy	*y* = 2562.2 × *x* + 1219	*y* = 111,690.4 × *x* + −54,521.533	0.995	0.963	244E + 02	0.999
DbalPy	*y* = 4827.537 × *x* + 228.287	*y* = 96,890.171 × *x* + −16,333.8	0.994	0.961	168E + 06	**0.367**
Phen	*y* = 690,114.966 × *x* + −4492.349	*y* = 351,650 × *x* + 81,455.4	0.970	0.994	143E + 02	0.999
I-Py	*y* = 4212.659 × *x* + −192.091	*y* = 119,493 × *x* + −41,462	0.990	0.955	297E + 05	0.936*
Naph	*y* = 1,134,412.925 × *x* + −6272.017	*y* = 397,360 × *x* + 74,348.6	0.988	0.972	798E + 03	0.9989

*5-MC* 5-methylchrysene, *BaA* benzo(a)anthracene, *BaP* benzo(a)pyrene, *BcF *benzo(c)fluorene, *BghiPer* benzo(g,h,i)perylene, *BjF* benzo(j)fluoranthene, *BkF* benzo(k)fluoranthene, *CCP* cyclopenta(c,d)pyrene, *Chry* chrysene, *DbaePy* dibenzo(a,e)pyrene, *DbahA* dibenzo(a,h)anthracene, *DbahPy* dibenzo(a,h)pyrene, *DbaiPy* dibenzo(a,i)pyrene, *DbalPy* dibenzo(a,l)pyrene, *Phen* phenanthrene, *I-Py* indeno(1,2,3-cd)pyrene, *Naph* naphthalene

**Bold text highlights analytes that failed to meet method validation criteria

The co-elution of BjF with BkF is noteworthy. Even monitoring programs using more powerful instrumentation, such as GC-MS/MS rather than a single quadrupole, run into the same limitation with this isomer group. The PAH4 and PAH8 indices endorsed by EFSA (2008), the regulatory standard for PAH assessment in food and biota, include benzo[k]fluoranthene as a diagnostic compound but omit benzo[j]fluoranthene entirely (EFSA, 2008), reflecting a field-wide acknowledgement that BjF is disproportionately difficult to resolve, but rarely changes environmental interpretation. Thus, excluding BjF and BkF herein does not put this dataset at a disadvantage relative to how comparable biomonitoring studies already report this isomer group. In fact, GC-based PAH biomonitoring in mussel tissue analyses routinely report 15 to 18 quantifiable compounds out of an original 18 to 26 compound panel (Grigoriou et al., 2021), indicating that partial quantifiability from co-elution or trace-level non-detection is typical and not a shortcoming specific to the DB-5MS column used here.

Two compounds, namely, benzo(k)fluoranthene (BkF) and BjF, share an identical retention time (40.944 min) and could not be resolved as separate signals. This co-elution is a documented limitation of 5%-phenyl methylpolysiloxane columns such as the DB-5MS used herein, with reliable reporting only possible at the level of summed benzofluoranthene isomers, typically requiring a mid-polar phase or tandem mass spectrometry for full resolution (Ziegenhals et al., 2008). Reported resolution values for this isomer pair on comparable columns fall as low as 1.41, well below what confident individual quantification requires, consistent with the complete overlap observed here. Both compounds were, therefore, excluded from quantitative interpretation. On the other hand, indeno(1,2,3-cd)pyrene (I-Py) yielded no quantifiable signal at any of the three fortification levels, falling below the instrumental detection capability of the method under the adopted SIM parameters, and was likewise excluded from all quantitative outputs and compositional indices.

#### Linearity

The GC-MS method developed for the determination of 15 of the 18 PAHs in mussel samples was validated using calibration curves constructed both in pure solvent and matrix, with linearity confirmed for most compounds (*r*^2^ > 0.97), except for benzo(j)fluoranthene (BjF), which presented an *r*^2^ of 0.809. The validation process also involved assessing other analytical parameters such as precision, accuracy, and limits of detection for each compound. Matrix-matched calibration curves were likely used to account for potential matrix effects that could influence PAH quantification in mussel samples. The lower linearity observed for benzo(j)fluoranthene suggests potential challenges in its quantification, possibly due to co-elution issues or matrix interferences specific to this compound.

#### Matrix effect

**Fig. 4:**
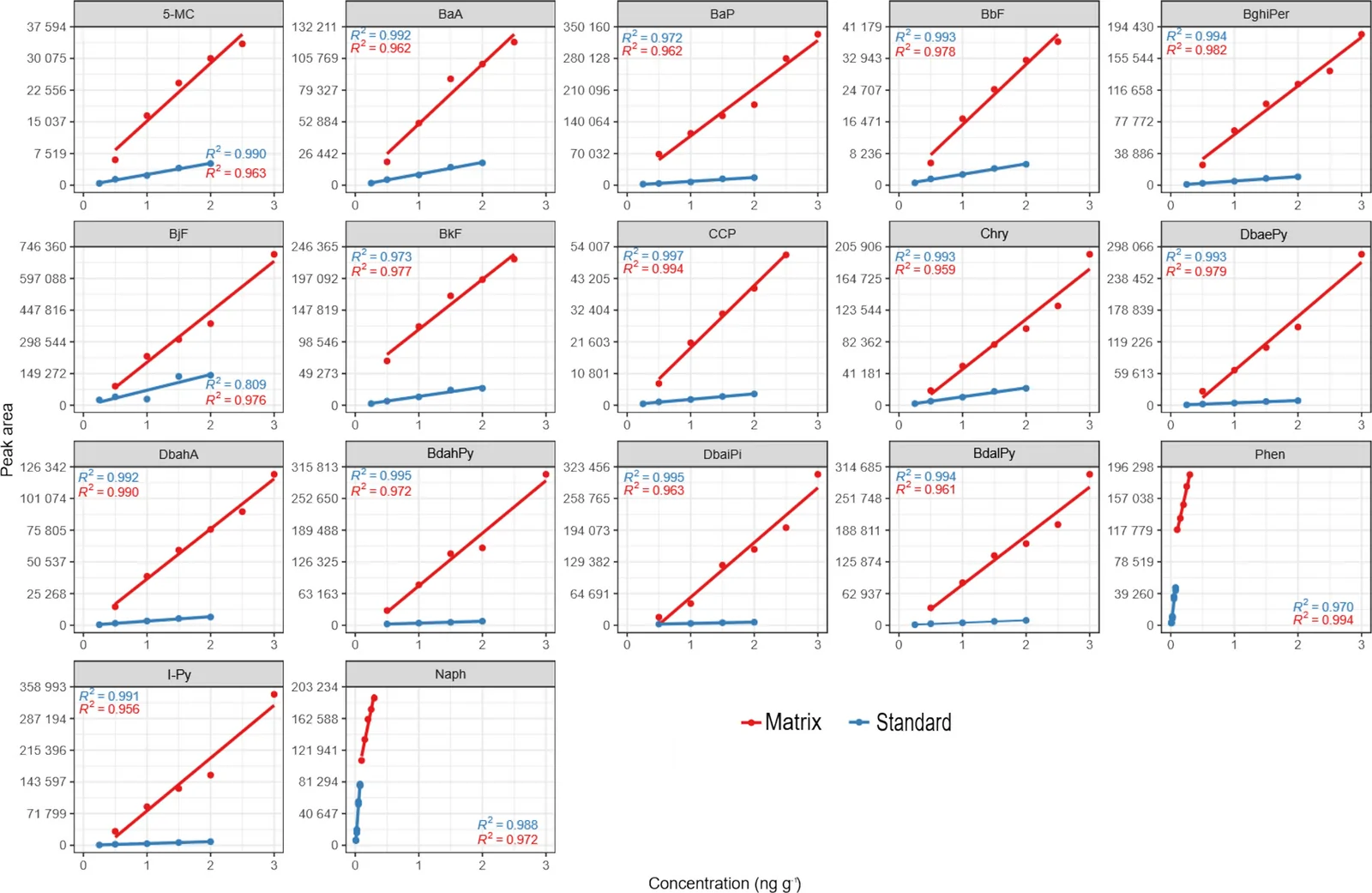
Calibration curves and regression analysis were performed for the quantification of polycyclic aromatic hydrocarbons (PAHs) in mussel (*Perna perna*) samples using gas chromatography-mass spectrometry (GC-MS). The red and blue lines represent the calibration curves for the matrix and standard solutions, respectively, across various PAHs, including Benzo(ghi)perylene (BghiPer), Dibenz(a,h)anthracene (DbaePi), Benzo(k)fluoranthene (BkF), Benzo(b)fluoranthene (BbF), and others. The *R*^2^ values indicate the goodness of fit for each calibration curve, demonstrating the linearity and reliability of the method under both matrix and standard conditions

Matrix effects were evaluated by comparing calibration curves built in pure solvent (dichloromethane) and in mussel matrix (Fig. 4), with an *F*-test applied to each compound to test for significant differences in slope and intercept between the two models (Table 2). No statistically significant difference was found between solvent and matrix curves for all PAH (*p* > 0.05 in all cases), including for HMW compounds such as BjF (*F* = 3.30 × 10^4^, *p* = 0.990) and low-molecular-weight (LMW) compounds such as 5-methylchrysene (*F* = 0.00798, *p* = 0.9988). For PAH analyses, GC-MS is often preferred over other techniques, such as liquid chromatography (LC), due to its superior selectivity, resolution, and sensitivity for PAH determination (Cha et al., 2018; Nácher-Mestre et al., 2009), allowing for the detection of PAHs at trace levels in complex matrices (van Drooge et al., 2023).

This range in *F*-values, from near zero to the order of 10^4^, while all converging on non-significant *p*-values, reflects differences in curve variance and degrees of freedom across compounds rather than inconsistent matrix behaviour, as the *p*-value, not the magnitude of *F*, is what determines statistical significance here, and by that criterion the calibration response was unaffected by matrix composition for every compound tested, including BghiPer and DbalPy, whose curves also showed no significant difference despite comparatively lower *p*-values (0.698 and 0.367) than the rest of the panel.

Despite this statistical equivalence, matrix-matched curves consistently showed slightly steeper slopes than their solvent-only counterparts across the PAH panel, a pattern suggestive of mild signal enhancement. Because this trend did not reach statistical significance for any individual compound, it likely reflects a low-level, systematic effect of the lipid-rich mussel matrix on ionization efficiency, rather than a compound-specific interference. Matrix-matched calibration was nonetheless retained as the quantification strategy, both as a conservative safeguard against this residual enhancement and because it avoids the assumption that solvent-based curves fully represent the extract's true chemical environment.

The absence of significant matrix effects, even for structurally complex, high-molecular-weight PAHs like BjF, benzo(a)pyrene, and dibenzo(a,h)anthracene, is a meaningful outcome given the challenges these compounds typically pose: they co-occur with abundant lipid material in mussel tissue, share similar hydrophobicity and retention behaviour, and are consequently prone to co-extraction with matrix interferents that can suppress or enhance their ionization. That this did not translate into a detectable shift in calibration response suggests the extraction protocol, particularly the 1:1 acetonitrile:dichloromethane mixture used to isolate lipophilic compounds, was effective at minimizing co-extracted lipid load ahead of GC-MS analysis.

#### Precision and recovery

Method precision, which included repeatability and intermediate precision (or reproducibility), was assessed by analyzing the relative standard deviation (RSD) between replicates (< 20%). Table 3 depicts each target PAH, detailing molecular masses and petrogenic/anthropogenic origins. For the majority of target compounds, mean recoveries fell within the 50–110% range across all three fortification levels, with RSDs below 20%, meeting the performance criteria established for environmental biomonitoring applications (USEPA, 2007). Petrogenic LMW compounds, including naphthalene and phenanthrene, exhibited consistent recoveries between 67.4 and 108.9% and RSDs below 16% across all fortification levels, reflecting the effective extraction of two- and three-ring PAHs by the ACN:DCM (1:1) solvent system.

**Table 3:** Identification of target polycyclic aromatic hydrocarbons in mussels from southeastern Brazil, detailing molecular masses and petrogenic/anthropogenic origins

Origin and persistence	PAH	Molecular weight (g mol^−1^)	Ions	Retention time	Fortification level (ng g^−1^)	Recovery (%)	RSD
Petrogenic Moderately persistent	Naph	128.17	*128*	6.625	1.0	78.204	8.975
					1.5	91.737	7.943
					2.0	108.972	8.219
Petrogenic Moderately persistent	Phen	178.23	178	14.084	1.0	67.365	12.672
					1.5	88.902	15.128
					2.0	102.221	11.246
Pyrogenic Highly persistent	BbF	216.28	216	24.259	1.0	53.111	14.368
					1.5	74.982	13.412
					2.0	99.108	2.898
Pyrogenic Moderately persistent	CCP	226.23	226	27.012	1.0	54.762	7.058
					1.5	73.484	12.702
					2.0	88.979	4.192
Moderately persistent	BaA	228.25	228	29.568	1.0	49.447	11.019
					1.5	73.207	15.041
					2.0	91.347	2.669
Moderately persistent	Chry	228.25	242	31.143	1.0	69.372	7.530
					1.5	75.889	10.758
					2.0	82.769	3.812
Petrogenic -	5-MC	228.25	252	35.421	1.0	50.408	11.717
					1.5	79.557	14.983
					2.0	102.160	6.437
Pyrolytic Moderately persistent	BjF	252.31	252	40.944	1.0	60.476	13.962
					1.5	65.629	15.046
					2.0	85.613	9.654
Pyrolytic Moderately persistent	BkF	252.32	276	40.944	1.0	23.914	63.785
					1.5	58.842	28.147
					2.0	98.177	14.984
Pyrolytic -	BaP	252.31	278	43.774	1.0	51.078	22.334
					1.5	57.901	22.211
					2.0	69.178	20.800
Pyrolytic Moderately persistent	Ipi	276.26	302	53.821	1.0	-	-
					1.5	-	-
					2.0	-	-
Pyrolytic Moderately persistent	DbahA	278.34	302	54.373	1.0	73.194	13.714
					1.5	78.315	13.501
					2.0	115.854	13.995
Pyrolytic	BghiPy		276	55.989	1.0	73.821	14.985
					1.5	77.084	15.180
					2.0	98.217	31.443
Pyrolytic -	DbaePy	276.26		66.000	1.0	78.527	9.143
					1.5	73.845	7.884
					2.0	88.459	1.901
Pyrolytic Moderately persistent	DbaiPy	276.26	302	69.041	1.0	96.665	7.817
					1.5	85.825	6.462
					2.0	101.597	3.079
Pyrolytic Moderately persistent	DbalPy	278.34	278	70.143	1.0	74.535	12.103
					1.5	72.007	8.441
					2.0	99.144	5.262
Pyrolytic Moderately persistent	DBahPy	278.34	302	70.755	1.0	77.488	9.620
					1.5	73.754	10.520
					2.0	92.716	2.299

Molecular weight ranges. Persistence: Low M.W < 180 g mol^−1^; Medium 180 > 250 g mol^−1^; High > 250 g mol^−1^. Low solubility < 0.1 mg L^−1^; Moderate 0.1 > 1 mg L ^−1^; High: > 1 mg L. Rapid persistence < 30 days; moderate persistence 30 < 180 days, high persistence > 180 days

*5-MC*, 5-methylchrysene; *BaA*, benzo(a)anthracene; *BaP*, benzo(a)pyrene; *BbF*, benzo(b)fluoranthene; *BghiPer*, benzo(g,h,i)perylene; *BjF*, benzo(j)fluoranthene; *BkF*, benzo(k)fluoranthene; *CCP*, cyclopenta(c,d)pyrene; *Chry*, chrysene; *DbaePy*, dibenzo(a,e)pyrene; *DbahA*, dibenzo(a,h)anthracene; *DbahPy*, dibenzo(a,h)pyrene; *DbaiPy*, dibenzo(a,i)pyrene; *DbalPy*, dibenzo(a,l)pyrene; *I-Py*, indeno(1,2,3-cd)pyrene; Naph, naphthalene; *Phen*, phenanthrene. *RSD*, relative standard deviation; *LOD*, limit of detection; *LOQ*, limit of quantification

Higher molecular mass compounds, such as BghiPer and DbaiP, exhibited distinct recovery and precision patterns, possibly due to their physicochemical properties or interactions with the sample matrix or the extraction method (Table 3). Similarly, PAHs derived from petrogenic (Naph and Phen) and pyrogenic (BaP and BkF) sources also varied among samples. The QuEChERS method has been extensively validated and proven effective for the analysis of various compounds, including PAH, pesticides, and other organic pollutants in matrices such as soil, sediment, seafood, and food products (Song et al., 2019).

Three compounds, however, exhibited performance outside the established criteria and warrant explicit discussion. Benzo(k)fluoranthene (BkF) presented an RSD of 63.8% at the lowest fortification level (1.0 ng g^−1^), decreasing to 28.1% and 15.0% at 1.5 and 2.0 ng g^−1^, respectively. This pattern of concentration-dependent precision is consistent with co-elution interference from benzo(j)fluoranthene (BjF), which shares an identical chromatographic retention time of 40.944 min under the present GC conditions, leading to signal overlap and variable quantification at trace concentrations. Both BkF and BjF are therefore excluded from quantitative interpretation in this study; their co-elution represents a known analytical challenge for five-ring fluoranthene isomers by GC-MS that typically requires either alternative column stationary phases or tandem mass spectrometry for full resolution (Grigoriou et al., 2021; Zelinková & Wenzl, 2015). Benzo(a)pyrene (BaP) presented RSDs between 20.8 and 22.3% across all fortification levels, marginally exceeding the 20% threshold. Given the consistency of this precision across concentrations and the environmental significance of BaP as a priority carcinogenic PAH, this compound was retained for quantitative reporting with the acknowledged caveat that its measurement uncertainty is slightly elevated relative to the method criterion. Benzo(g,h,i)perylene (BghiPer) showed acceptable recoveries at the 1.0 and 1.5 ng g^−1^ levels but an RSD of 31.4% at 2.0 ng g^−1^, attributed to residual lipid matrix interference at higher analyte concentrations, consistent with the elevated F-value observed for this compound in the matrix effect assessment (Table 2). Finally, indeno(1,2,3-cd)pyrene (I-Py) yielded no quantifiable signal at any of the three fortification levels, indicating that this compound falls below the instrumental detection capability of the present method under the adopted SIM acquisition parameters. I-Py is accordingly excluded from all quantitative outputs and compositional indices reported in this study.

#### Limits of detection and limits of quantification

Method LODs and LOQs for the 15 quantifiable PAHs are presented in Table 4, covering instrumental ranges from 0.009 to 1.147 ng g^−1^ and 0.028 to 3.477 ng g^−1^, respectively. These limits reflect the sensitivity of the QuEChERS-SPE-GC-MS approach applied to lipid-rich mussel tissue and are appropriate for the detection of PAH contamination at environmentally relevant concentrations in the context of coastal biomonitoring, consistent with WHO analytical quality recommendations for environmental monitoring programs (WHO, 1995). The lowest LODs were obtained for naphthalene (0.009 ng g^−1^) and phenanthrene (0.015 ng g^−1^), reflecting the higher GC-MS sensitivity for LMW PAHs with less complex fragmentation patterns. In contrast, benzo(j)fluoranthene exhibited the highest LOD (1.147 ng g^−1^), consistent with its poor linearity discussed in Sect. "Selectivity", and was therefore excluded from quantitative interpretation. Indeno(1,2,3-cd)pyrene could not be quantified at any of the three fortification levels and is similarly excluded from quantitative reporting. The method LODs obtained for the remaining compounds are comparable to those reported by Grigoriou et al. (2021) for *Mytilus galloprovincialis* using GC-MS/MS and by Pfannkoch et al. (2015) for QuEChERS-based PAH determination in oysters, supporting the analytical adequacy of the present method for marine bivalve biomonitoring applications.

**Table 4:** Limits of detection (LOD) and limits of quantification (LOQ) for polycyclic aromatic hydrocarbons in mussel tissues from southeastern Brazil using a modified QuEChERS-GC/MS Analysis

Compound	Instrumental range		Method range	
	LOD (ng g^−1^)	LOQ (ng g^−1^)	LOD (ng g^−1^)	LOQ (ng g^−1^)
5-MC	0.236	0.716	0.514	1.559
BaA	0.210	0.637	0.522	1.581
BAP	0.398	1.206	0.616	1.869
BbF	0.200	0.608	0.387	1.174
BGHIPER	0.191	0.579	0.421	1.277
BjF	1.147	3.477	0.497	1.507
BKF	0.391	1.185	0.398	1.207
CCP	0.125	0.379	0.208	0.632
Chry	0.198	0.601	0.639	1.936
DbaePy	0.205	0.621	0.465	1.409
DbahA	0.216	0.654	0.310	0.938
DbahPy	0.156	0.473	0.536	1.625
DbaiPy	0.158	0.479	0.604	1.830
DbalPy	0.188	0.571	0.621	1.880
Phen	0.015	0.045	0.021	0.062
I-Py	0.232	0.702	0.683	2.071
Naph	0.009	0.028	0.045	0.134

The LOD for benzo(a)anthracene found in this study was 0.210 ng g⁻^1^ in the instrumental range, with an LOQ of 0.637 ng g^−1^. These values are higher than those reported by Grigoriou et al. (2021) for the same compound in *Mytilus galloprovincialis* (LOD = 0.006 ng g^−1^; LOQ = 0.02 ng g^−1^), reflecting their use of isotope-dilution GC-MS/MS rather than the matrix-matched, single-quadrupole GC-MS approach used here. Pfannkoch et al. (2015) similarly demonstrated the effectiveness of QuEChERS-based extraction for PAH analysis in seafood, including oysters and mussels, though paired with stir bar sorptive extraction (SBSE) rather than SPE clean-up, achieving recoveries of 65–138%.

On the other hand, the LODs and LOQs for benzo(a)pyrene (BaP) in this study (Table 4) were higher than the sub-picogram-per-microliter quantification limit reported by van Drooge et al. (2023) using GC-Orbitrap-MS for airborne PAH monitoring; this comparison should be read with caution, however, as their limit is expressed per microliter of injected extract in atmospheric particulate matter, not per unit mass of sample matrix, and is therefore not directly convertible to the ng g⁻^1^ basis used here. Despite this difference in instrumentation and matrix, the results presented herein remain within the acceptable limits established by the European Union, as previously discussed.

Zelinková and Wenzl (2015) reported LOQs ranging from 0.05 to 0.25 ng g^−1^ across a panel of 32 PAHs, including methylated homologues, in food supplements, with recoveries of 73–97%, values broadly comparable to those obtained in the present study for most target compounds. Reduced recovery for BkF specifically has also been documented elsewhere: in a validated SBSE-HPLC-FLD method for priority PAHs in surface water, BkF showed a comparatively low mean recovery of 66.5%, attributed to matrix effects from higher total suspended solids and organic matter content relative to cleaner water matrices.

### Distribution of ∑PAH and the sampling locations, status, and seasonality

The analytical performance demonstrated provided the methodological foundation necessary to interpret low or absent PAH signals as genuine environmental results rather than analytical artefacts. Of the 18 PAHs targeted, concentrations above the LOD were detected in only five compounds across the 210 mussel samples analysed, with most measurements falling below the LOD. This pattern of low detection, rather than representing a methodological limitation, reflects the biomonitoring reality of the studied coastal area at the time of sampling, about 2 years after the 2019 oil spill, and is consistent with evidence of natural attenuation of oil-derived contaminants in tropical marine environments over medium-term timescales (Nunes et al., 2023; Pereira et al., 2023).

**Fig. 5:**
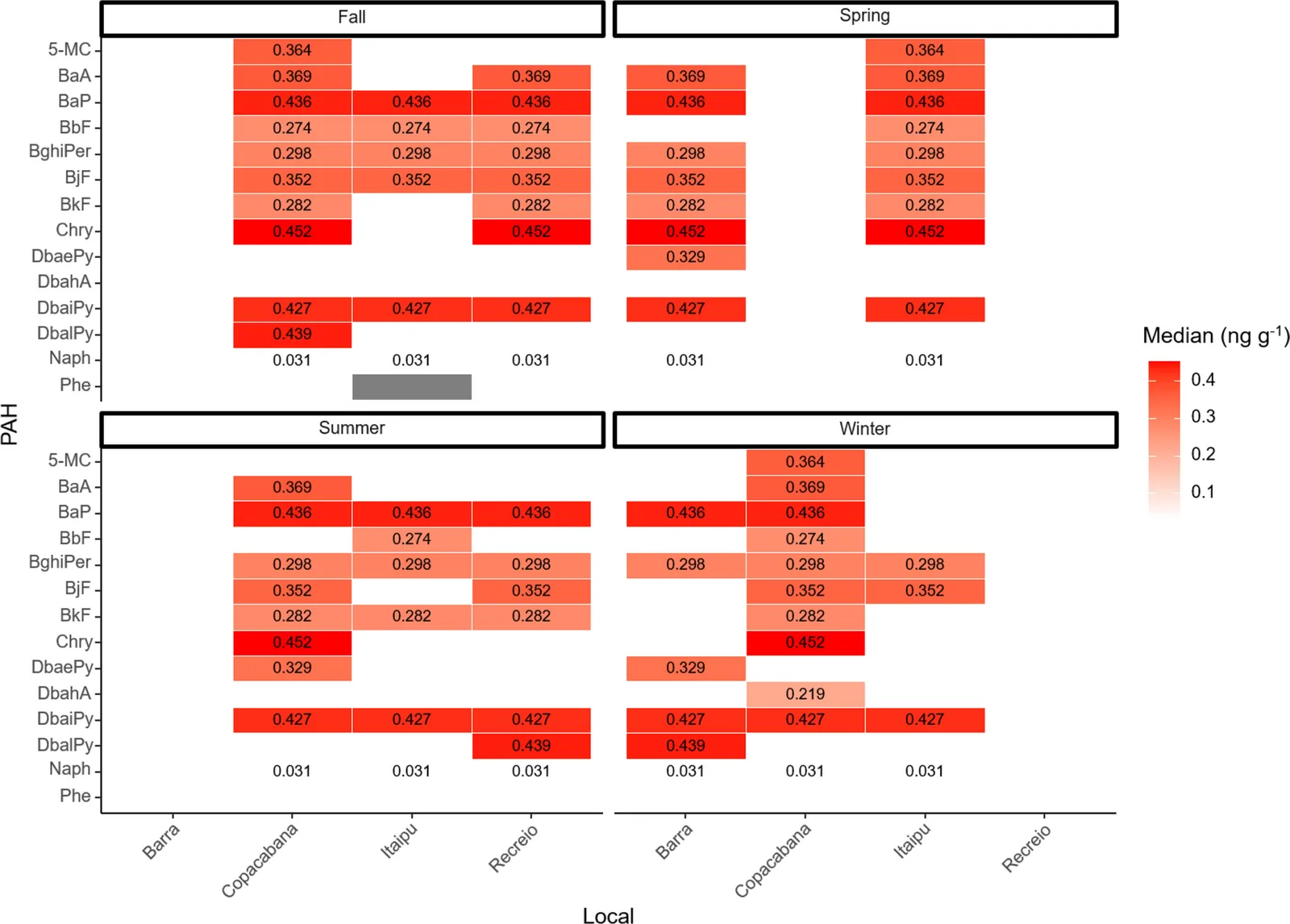
Imputed data obtained after statistical treatment. Values are represented as medians, given that the data did not follow a normal distribution. Note that, for several sites and sampling dates, mean values were higher than the quantification limit (LQ)

To address the high proportion of censored observations, values below the LOD were replaced by LOD/2, following the imputation strategy recommended by the World Health Organization for environmental datasets with non-detects (WHO, 1995). This approach minimizes systematic bias in the estimation of central tendency metrics and is widely applied in studies of organic contaminants in marine matrices (Helsel, 2006; Menzie et al., 2002; Yunker et al., 2002). Following imputation, spatial and temporal concentration patterns became discernible across sampling sites and seasons, as presented in Fig. 5, though overall PAH burdens remained low throughout the dataset. After that, spatial and temporal compound differences become evident, although most PAH concentrations remain low. Similar patterns have been reported in marine and coastal environments, where seasonal variations and site-specific inputs contribute to heterogeneous PAH distributions (Boonyatumanond et al., 2006; Perra et al., 2011).

Five PAHs were detected above the LOD across the 210 mussel samples following imputation, with concentrations varying across sampling sites and seasons. 5-Methylchrysene (5-MC) was detected in Copacabana (winter and autumn) and Itaipu (spring) at a mean concentration of 0.257 ng g^−1^, while benzo(a)anthracene (BaA) was quantified across all four sites at around 0.261 ng g^−1^. Benzo(a)pyrene (BaP) reached the highest mean concentration among detected compounds (0.308 ng g^−1^) and was present across all sampling locations and seasons, suggesting continuous rather than episodic contamination inputs. The Kruskal–Wallis test confirmed no statistically significant differences in PAH concentrations across sites (*p* = 0.492) or seasons (*p* = 0.140), indicating spatial and temporal homogeneity in spatial and temporal contamination levels during the monitoring period, though this absence of significant differences may also reflect reduced statistical power due to the high proportion of non-detects and LOD/2 imputation rather than true source stability, consistent with findings by Degger et al. (2011) for chronically impacted coastal systems.

The toxicological significance of the detected compounds warrants explicit discussion, as the five PAHs identified represent some of the most hazardous compounds within this chemical class. BaP is the only PAH classified by the International Agency for Research on Cancer (IARC) as a confirmed human carcinogen (Group 1), acting primarily through metabolic activation to reactive diol-epoxide intermediates that form covalent DNA adducts. This mechanism is well documented in mussels, for instance, BaP exposure has been shown to induce digestive gland biotransformation enzymes and cause measurable DNA damage in *Mytilus galloprovincialis* under both acute laboratory exposure and field conditions (Banni et al., 2010; Canova et al., 1998). Benzo[a]pyrene hydroxylase (BPH) activity, reflecting CYP1A induction, is consequently established as a standard molecular biomarker of PAH exposure in mussel digestive gland tissue (Banni et al., 2010). In *Perna perna* specifically, PAH exposure and its biological effects have been directly evaluated in populations from Rio de Janeiro, providing local precedent for interpreting biomarker and tissue-burden data in this species (Francioni et al., 2007a), whilst simultaneously generating toxic quinone and phenol metabolites that promote oxidative stress and lipid peroxidation at sub-lethal concentrations (Banni et al., 2010). BaA and 5-MC are both classified as possible human carcinogens (IARC Group 2B), with mutagenic activity demonstrated in multiple aquatic model organisms (Hatami Manesh et al., 2021). The detection of these compounds in mussels consumed by artisanal fishing communities represents a chronic low-dose exposure scenario that, while below acute toxicity thresholds, is particularly relevant given the cumulative nature of PAH carcinogenicity and the absence of a recognised safe threshold for genotoxic compounds (Perugini et al., 2007).

The predominance of BaP and BaA, both pyrogenic markers strongly associated with fossil fuel combustion and urban atmospheric deposition, over petrogenic LMW compounds such as naphthalene and phenanthrene, supports the hypothesis that diffuse atmospheric sources, including vehicle emissions, industrial combustion processes and harbour traffic, constitute the primary contamination pathway in the studied coastal areas (Cabral et al., 2022; Taniguchi, 2001). This pyrogenic signature contrasts with what would be expected from direct petroleum spill inputs, which typically produce naphthalene- and phenanthrene-dominated petrogenic profiles (Yoshimine et al., 2012). The absence of significant seasonal variation in PAH concentrations further supports a diffuse, continuous atmospheric deposition model rather than point-source inputs, which typically produce acute concentration peaks associated with specific events and show stronger temporal variation (Webster & Kott, 2011). Point-source contributions from vessel traffic and harbour activities within and adjacent to GB may nonetheless represent a secondary, chronic petrogenic input, as suggested by the detection of naphthalene and phenanthrene, both characteristic both pyrogenic and petrogenic sources, at Copacabana and Barra da Tijuca, sites with higher recreational and commercial maritime activity.

The detection of BaA and 5-MC specifically, rather than the full PAH panel, in spring samples from Itaipu and Copacabana is worth interpreting considering what is known about these two compounds individually, rather than framed as evidence of a seasonal physiological effect that was not directly measured here. Both are combustion-derived markers, the diagnostic ratio BaA/(BaA + Chr), when above 0.35, is a recognized indicator of combustion and vehicular emission sources rather than petrogenic input (Tobiszewski & Namieśnik, 2012; Zeng & Vista, 1997), and 5-MC is one of the 15 EU priority PAHs flagged for combined genotoxic and carcinogenic concern, alongside BaP, based on its metabolic activation to potent tumorigenic dihydrodiol-epoxide intermediates. Their co-occurrence in spring samples is therefore more parsimoniously read as reflecting a seasonal pulse in combustion-derived input reaching these sites (e.g., increased urban runoff, traffic, or atmospheric deposition), rather than seasonal variation in the mussels' own biotransformation capacity, which was not assessed in this study. Given the relatively short biological half-life of PAHs in marine bivalves generally (2–16 days; Eisler, 1987), tissue burden at the time of sampling likely reflects recent, rather than cumulative, exposure. Future studies should, thus, pair seasonal sampling with direct CYP1A biomarker determinations to distinguish between both.

Comparing detected concentrations with international reference values, BaP at 0.308 ng g^−1^ remains below the European Union maximum level of 5 ng g^−1^ for processed bivalve molluscs and the PAH4 limit of 30 ng g^−1^ (European Commission, 2011). Total PAH burdens in the present study are substantially lower than the 60–6271 ng g^−1^ reported for *Perna perna* from Guanabara Bay in earlier baseline assessments (Francioni et al., 2007a, b), and below the 591–997 ng g^−1^ range reported for mussels from Jakarta Bay by Aziz et al. (2021). Bivalves exhibiting total PAH burdens between 300 and 500 ng g^−1^ are generally considered to represent low-level contamination (Kasiotis & Emmanouil, 2015), a threshold not exceeded in the present dataset. Nonetheless, the chronic nature of exposure to genotoxic compounds such as BaP, even at concentrations below acute effect thresholds, justifies continued biomonitoring efforts, particularly given the sustained atmospheric deposition from urban and industrial sources along the GB coastal zone and the dependence of local communities on mussel consumption as a primary protein source (Francioni et al., 2007b; Olatunji, 2020).

The cooking status of samples (fresh versus pre-cooked) did not significantly alter the detected PAH profile, consistent with the observation that boiling and steaming, methods commonly employed by artisanal communities in this region, may partially reduce PAH concentrations by leaching into cooking water but have limited efficacy when initial tissue burdens are low (Sobral et al., 2020). In contrast, high-temperature preparation methods such as grilling or frying are known to generate additional PAH through pyrolytic reactions, potentially increasing consumer exposure beyond the levels measured in raw tissue (Sobral et al., 2020). This distinction is particularly relevant for the artisanal fishing communities surrounding GB, for whom smoked and grilled preparations are culturally common.

The ΣPAHs, calculated as the mean of the per-sample sums over the 15 validated analytes with non-detected compounds substituted by LOD/2, was 4.27 ng g^−1^, dominated by the HMW fraction (ΣHMW = 4.24 ng g^−1^) and with a negligible LMW contribution (ΣLMW = 0.03 ng g^−1^). Because censored values contribute to this total, ΣPAHs is not directly comparable with the per-compound means reported above, which refer only to detected values. The compositional pattern is consistent with chronic atmospheric pyrogenic deposition rather than acute petrogenic contamination. Although formal health risk indices such as Estimated Daily Intake (EDI), Hazard Quotient (HQ), Toxic Equivalency (TEQ) and Incremental Lifetime Cancer Risk (ILCR) were not calculated given that measured concentrations were below the method LOQ, the qualitative risk profile indicated by the compound composition, with BaP, BaA, and 5-MC as dominant contributors highlights the importance of sustained biomonitoring of pyrogenic PAHs in *P. perna* from this coastline, independently of acute regulatory exceedances.

Here, any interpretation is limited by the lack of pre-spill data for these sites and of an uncontaminated reference area. The gap is not particular to this dataset: on the adjacent Rio de Janeiro coast, PAH burdens in *P. perna* could only be set against scattered assessments from the previous decade, which is itself an argument for sustained, long-term biomonitoring (Carreira & Gramlich, 2025). Low burdens therefore cannot be read as recovery from the 2019 spill, nor can the spill be separated from chronic urban-industrial inputs.

Taken together, a validated method applied to the 210 mussel samples returned only five PAHs above the LOD, all well below EU maximum levels and homogeneous across sites and seasons, but dominated by combustion-derived, high-molecular-weight compounds rather than by petroleum residues. At first, this is a counterintuitive profile for a bay whose contamination history has been dominated by refineries, terminals and repeated oil spills, but it is consistent with recent evidence from the Rio de Janeiro coast, where combustion sources tied to urban expansion contribute to the PAH burden of *P. pern*a alongside petrogenic inputs (Carreira & Gramlich, 2025; Ceccopieri et al., 2023). Since BaP, BaA, and 5-MC have no recognised safe exposure threshold and mussels remain a staple protein locally, the argument for continued surveillance rests on which compounds are present, not on how much of them.

## Conclusions

The proposed QuEChERS-SPE-GC-MS method was successfully validated for 15 of the 18 target PAHs, achieving recoveries between 51.1 and 109.0% and satisfactory linearity (r^2^ > 0.95) across the validated concentration range. Benzo(j)fluoranthene (BjF) and benzo(k)fluoranthene (BkF) were excluded due to chromatographic co-elution, while indeno(1,2,3-cd)pyrene (I-Py) remained below the method LOD throughout validation. Reporting these analytical limitations provides a transparent assessment of the method’s capabilities and constraints, facilitating its appropriate application in future monitoring studies. Combined with matrix-matched calibration instead of isotope dilution and a solvent-efficient QuEChERS-SPE extraction protocol, the method offers a practical and cost-effective alternative for routine biomonitoring of PAHs in mussel tissues, particularly in laboratories with limited analytical resources.

Application of the validated method to mussels collected from artisanal fishing communities in Guanabara Bay, approximately 2 years after the 2019 oil spill, resulted in the detection of naphthalene, phenanthrene, 5-methylchrysene, benzo(a)anthracene, and benzo(a)pyrene. These findings should be, however, interpreted cautiously, as the absence of pre-spill baseline data or an uncontaminated reference site precludes definitive conclusions regarding environmental recovery. Nevertheless, the predominance of high-molecular-weight PAHs, reflected by the compositional indices (ΣPAHs and ΣHMW), suggests that current contamination is more consistent with pyrogenic inputs associated with fossil fuel combustion, atmospheric deposition, and urban traffic than with residual petroleum contamination from the spill.

## Data Availability

The datasets generated and/or analyzed during the current study, including biomonitoring data and contaminant concentrations in fish samples, are available from the corresponding author upon reasonable request.
